# Depletion of T cells *via* Inducible Caspase 9 Increases Safety of Adoptive T-Cell Therapy Against Chronic Hepatitis B

**DOI:** 10.3389/fimmu.2021.734246

**Published:** 2021-10-06

**Authors:** Alexandre Klopp, Sophia Schreiber, Anna D. Kosinska, Martin Pulé, Ulrike Protzer, Karin Wisskirchen

**Affiliations:** ^1^ School of Medicine, Institute of Virology, Technical University of Munich, Munich, Germany; ^2^ Institute of Virology, Helmholtz Zentrum München, Munich, Germany; ^3^ German Center for Infection Research (DZIF), Munich Partner Site, Munich, Germany; ^4^ Department of Haematology, Cancer Institute, University College London, London, United Kingdom

**Keywords:** T-cell therapy, chronic hepatitis B, safeguard molecules, inducible caspase 9, suicide switch, chimeric antigen receptor (CAR)

## Abstract

T-cell therapy with T cells that are re-directed to hepatitis B virus (HBV)-infected cells by virus-specific receptors is a promising therapeutic approach for treatment of chronic hepatitis B and HBV-associated cancer. Due to the high number of target cells, however, side effects such as cytokine release syndrome or hepatotoxicity may limit safety. A safeguard mechanism, which allows depletion of transferred T cells on demand, would thus be an interesting means to increase confidence in this approach. In this study, T cells were generated by retroviral transduction to express either an HBV-specific chimeric antigen receptor (S-CAR) or T-cell receptor (TCR), and in addition either inducible caspase 9 (iC9) or herpes simplex virus thymidine kinase (HSV-TK) as a safety switch. Real-time cytotoxicity assays using HBV-replicating hepatoma cells as targets revealed that activation of both safety switches stopped cytotoxicity of S-CAR- or TCR-transduced T cells within less than one hour. *In vivo*, induction of iC9 led to a strong and rapid reduction of transferred S-CAR T cells adoptively transferred into AAV-HBV-infected immune incompetent mice. One to six hours after injection of the iC9 dimerizer, over 90% reduction of S-CAR T cells in the blood and the spleen and of over 99% in the liver was observed, thereby limiting hepatotoxicity and stopping cytokine secretion. Simultaneously, however, the antiviral effect of S-CAR T cells was diminished because remaining S-CAR T cells were mostly non-functional and could not be restimulated with HBsAg. A second induction of iC9 was only able to deplete T cells in the liver. In conclusion, T cells co-expressing iC9 and HBV-specific receptors efficiently recognize and kill HBV-replicating cells. Induction of T-cell death via iC9 proved to be an efficient means to deplete transferred T cells *in vitro* and *in vivo* containing unwanted hepatotoxicity.

## Introduction

Worldwide, about 257 million humans are chronically infected with the hepatitis B virus (HBV) (1). Around one third of those will develop secondary diseases such as liver cirrhosis or hepatocellular carcinoma. The currently available treatments for chronic hepatitis B suppress viral replication but in most of the cases do not lead to eradication of the virus (2). In acute hepatitis B a strong immune response develops, in which CD8^+^ T cells are key to clearing the virus. By contrast, the T-cell response against the virus in patients with chronic hepatitis B is inefficient because it is weak and oligoclonal (3). Hence, adoptive T-cell therapy with HBV-specific T cells represents a promising therapeutic strategy in order to eliminate HBV and induce functional cure (4).

T cells can be genetically engineered to express either an HBV-specific chimeric antigen receptor (S-CAR) (5, 6) or a natural T-cell receptor (TCR) (7, 8). The S-CAR contains an antibody fragment that is directed against HBV surface proteins present on cells, in which the virus replicates (9). Upon binding to HBsAg on cells, the S-CAR T cell becomes activated through its CD28 and CD3 signaling domains. The natural TCRs recognize either HBV core- or S-peptides presented on HLA-A2 and their engagement leads to physiological activation of the T cell (7, 8). Previous *in vitro* experiments in our laboratory have demonstrated that both, S-CAR T cells and TCR T cells are very efficient effector cells, capable of eliminating up to 100% of HBV-replicating target cells and secreting high amounts of antiviral cytokines such as IFN-γ (5–7). *In vivo*, specific killing of HBV-replicating hepatocytes leads to a transient and moderate increase of alanine amino transferase (ALT) levels after transfer of S-CAR T cells into HBV-transgenic (6) or TCR T cells into HBV-infected uPA-SCID mice (8).

Since adoptive transfer of functionally active HBV-specific T cells intends to eliminate infected hepatocytes, the benefits of resolving persistent HBV infection may come along with risks of inducing a hepatitis flare. Furthermore, although S-CAR T cells only seem to be sensitive to cell- or plate-bound HBsAg *in vitro* and did not cause any severe liver damage *in vivo* (5, 6), soluble HBsAg in the serum of infected patients still poses a theoretical risk of off-site activation of the cells and an unwanted cytokine release. Hence, the evaluation of strategies to increase the safety of adoptively transferred HBV-specific T cells is an important step to prevent and overcome these potential threats.

Several clinical cancer trials using T-cell therapy have reported a morbidity and occasionally mortality resulting from T-cell toxicity (10). These include on-target, off-tumor effects because some target antigens are also expressed on healthy tissues (11, 12), or off-target effects due to unintentionally or deliberately (e.g. to increase affinity) inserted mutations in the sequence of a particular TCR or CAR (13–15). The most frequent side effect observed after CAR T-cell therapy is a cytokine release syndrome (CRS), which is believed to be an indirect consequence of an excessive expansion and activation of CAR T cells. It may even have a fatal outcome for the respective patient (16, 17), although clinicians in the meantime have gained ample experience how to handle CRS and prevent severe consequences (16).

To increase safety of adoptive cell transfer, various safeguard systems have been developed to allow depletion of transferred T cells on demand. Safeguard mechanisms can be expressed either intra- or extracellularly and, when activated, induce “suicide” of the transferred T cell. The most commonly tested mechanisms in the clinics are an inducible caspase 9 (iC9) (18) and a thymidine kinase of herpes simplex virus (HSV-TK) (19). iC9 consists of a part of the human caspase 9 and a domain that can bind the small molecules AP1903 and AP20187 with high affinity. These molecules act as chemical inducers of dimerization (CID) of the iC9 which then rapidly activates downstream executing caspases resulting in apoptosis of the cell carrying iC9. HSV-TK phosphorylates the prodrug ganciclovir (GCV) that is then incorporated into host DNA and terminates the elongation of DNA strands leading to cell death (20). Its effect, however, is not strictly limited to the HSV-TK positive cells and can also affect neighbouring cells (21).

In this study, we expressed either iC9 or HSV-TK in HBV-specific-TCR and S-CAR T cells. We investigated the effect of induction of these safeguard mechanisms on cytotoxicity and cytokine release of redirected T cells and the consequences of T-cell depletion for the antiviral effect *in vitro* in cell culture. Furthermore, depletion of S-CAR T-cells via iC9 was studied *in vivo* in a preclinical model of chronic HBV infection.

## Materials and Methods

### Cloning

All constructs (HBV-specific receptors and safeguard molecules) used were codon-optimized. iC9 or HSV-TK were linked to the respective receptors via a T2A element. The HBV-specific receptors were amplified from constructs previously described for the S-CAR (5) or the HBV-specific TCRs (7). Plasmids coding for the safeguard mechanisms [iC9 and HSV-TK (22)] were obtained from Martin Pulé. PCRs of safeguard and receptor genes were performed with the Phusion Hot Start Flex 2x Master Mix (New England Biolabs) according to the manufacturer’s instructions. The primers linking both fragments were designed with an overlap of ~ 18 bp. The fusion of two equimolar PCR fragments (total of 300 ng) was performed with 15 initial cycles without the primers followed by additional 15 cycles after addition of the primers. Plasmids were purified with the GeneJET Plasmid Miniprep Kit (Thermo Fisher Scientific) or the Plasmid *Plus*Midi Kit (Qiagen) depending on the desired quantity of purified plasmid, following the manufacturer’s instructions.

### Retroviral Transduction of Human PBMCs

24-well non-tissue culture plates (Falcon) were coated with human anti-CD3 (eBioscience) (5 μg/mL) and anti-CD28 (eBioscience) (0.05 μg/mL) antibodies (2 hours, 37°C) and blocked for 30 minutes at 37°C with 2% bovine serum albumin (BSA) in PBS. Freshly isolated or thawed human PBMCs were counted and adjusted to 0.6-0.8x10^6^ cells/mL human T cell medium (hTCM) [RPMI, 10% fetal calf serum (FCS), 1% glutamine, 1% penicillin and streptomycin, 1% sodium pyruvate, 1% non-essential amino acids (NEAA) and 0.01 M HEPES (all from Thermo Fisher Scientific)] supplemented with 300 U/mL IL-2 (Novartis) and 1.5 mL of the cell suspension was seeded per well (day one). Cells were incubated for 48 hours at 37°C. After incubation, two transduction rounds on two consecutive days (day three and four) were performed. Before transduction, non-tissue 6- or 24-well-plates were coated with RetroNectin (20 μg/mL in PBS, 2 hours at RT; Takara) and blocked for 30 minutes at 37°C with 2% BSA in PBS. Retroviral supernatants were obtained from RD114 packaging cells (293GP-R30 (23), Biovec pharma) that had been transfected (Lipofectamine 2000 from Invitrogen) with MP71 retroviral plasmids [as previously described (7)] containing the respective sequences, filtered (0.45 μm sterile filter from Sarstedt) and added to the wells. Plates were centrifuged (2000 g, 2 hours, 32°C). During centrifugation, the PBMCs were counted, washed and adjusted to 1x10^6^ cells per mL fresh hTCM supplemented with 300 U/mL IL-2. After centrifugation, the cell suspension was added to the plates containing the supernatants. The plates were centrifuged once again (1000 g, 10 minutes, 32°C) and incubated at 37°C overnight. On day five and six, the T cells were collected, washed and counted. They were then adjusted to 0.5x10^6^ cells/mL supplement with 180 U/mL IL-2 for expansion. On day ten, the transduction rate of the T cells was determined by flow cytometry (see below) and they were either frozen or subsequently used for functional assessment.

### Co-Culture Experiments

For functionality assessment, transduced T cells were co-cultured with target cells. For all co-culture experiments, unless otherwise stated, 5x10^4^ HepG2.2.15 cells [stable HBV-producing human hepatoma cell line derived from HepG2 (24, 25)] were seeded in 200 μL DMEM full medium (10% FCS, 1% penicillin and streptomycin, 1% glutamine, 1% NEEA and 1% sodium pyruvate) per well on a collagen-coated (Collagen R (Serva) 1:10 in H_2_O for 30 minutes at 37°C) 96-well electronic microtiter plates (ACEA Biosciences) seven to ten days prior start of the co-culture. The following day, medium was changed to Diff medium [DMEM full medium + 2.5% DMSO (Sigma)], allowing the differentiation of target cells. Transduced T cells were either taken from an expansion culture or thawed one day prior to starting the co-culture and kept in culture with 30 U/mL IL-2. On the day of the co-culture, transduced T cells were washed and resuspended in the appropriate amount of fresh hTCM without IL-2. Medium was changed on the plates with target cells to DMEM full medium with 2% DMSO, 100 μL/well. Transduced T cells were added to the wells in an indicated E:T ratio in 100 μL/well (final concentration of 1% DMSO in co-culture). The number of effector T cells/well was adjusted according to the transduction efficiency of each receptor to identical numbers of receptor-expressing cells. Real-time viability of target cells was determined using an xCELLigence SP Real-Time Cell Analyzer (ACEA Biosciences) allowing the quantitative and continuous monitoring of adherent target cells, through the measurement of electrical impedance every 15 minutes. The electrical impedance displayed as cell index (CI) and determining the target cell viability was normalized to the start of the co-culture. Experiments were performed in triplicates. For addition of CID [B/B Homodimerizer (AP20187) from Takara; 0.5 mM in 100% ethanol] or ganciclovir (Invivogen; 10 mg/mL in distilled water adjusted with NaOH 1M and HCl 1M to pH 11), 100 μL medium was removed per well. The respective substance was diluted in DMEM full medium to achieve the desired concentration and 100 μL were added per well.

The concentration of human IFN-γ in the supernatant to assess the activation of T cells was determined using MaxiSorb ELISA 96-well plates (Thermo Fisher Scientific) and commercially available ELISA kits (Invitrogen) according to manufacturer’s instructions. The conversion of TMB substrate was determined by measurement of OD_450_ subtracted by OD_560_ on an ELISA-Reader infinite F200 (Tecan).

### Flow Cytometry

For the staining of surface proteins, cells were resuspended in FACS buffer (0.1% BSA in PBS) with the respective antibodies and incubated for 30 minutes on ice and in the dark. The following antibodies were used: mCD4 (eBioscience), mCD8 (BD Biosciences), mCD45.1 (eBioscience), mCD45.2, mIFN-γ, mTNF-α (all from BD Biosciences); hCD4 (Thermo Fisher Scientific), hCD8 (Dako). Viability of cells was determined using a live/dead cell marker (LIVE/DEAD Fixable Green Dead Cell Stain Kit, Thermo Fisher Scientific). For intracellular cytokine staining, cells were permeabilized by resuspending them in Cytofix/Cytoperm (BD Biosciences) prior to the antibody staining, following the manufacturer’s instructions. In order to determine the absolute cell count by flow cytometry, CountBright™ Absolute Counting Beads (Thermo Fisher Scientific) were added to the cell suspension shortly before measurement. If CAR expression was analyzed together with other surface proteins such as CD8, stainings were performed sequentially. The CAR was first stained with an anti-human IgG antibody (Abcam) followed by the staining of the other surface proteins with the respective antibody. The samples were then analyzed on a CytoFLEX S flow cytometer (Beckman Coulter). The obtained data was evaluated with FlowJo 10.4.

### Retroviral Transduction of Murine Splenocytes

24-well non-tissue culture plates were coated with murine anti-CD3 and anti-CD28 antibodies (kindly provided by R. Feederle, Helmholtz Zentrum München) at a concentration of 10 μg/mL each in PBS for 2 hours at 37°C and blocked for 30 minutes at 37°C with 2% BSA in PBS. Freshly isolated murine splenocytes from donor mice were enriched for CD8^+^ by magnetic activated cell sorting (MACS) using CD8a (Ly2) Microbeads (Miltenyi). CD8^+^ T cells were separated according to manufacturer’s instructions. They were counted, washed and adjusted to 0.8x10^6^ cells per 1.5 mL mouse T cell medium (mTCM) (RPMI Dutch modified, 10% FCS, 1% glutamine, 1% penicillin and streptomycin, 1% sodium pyruvate, and 50 μM β-mercaptoethanol all from Thermo Fisher Scientific) and supplemented with 5 ng/mL IL-12 (kindly provided by E. Schmitt, University of Mainz). 1.5 mL of the cells were then seeded on the antibody-coated 24-well plates and incubated at 37°C overnight. On the following day, retroviral supernatant from Platinum-E packaging cells (based on the 293T cell line) transfected with MP71 retroviral plasmids containing the respective coding sequence was collected and filtered (0.45 μm sterile filter). Protamine sulfate (2 μg/mL; Leo Pharma) was added to the activated CD8^+^ T cells and the retroviral supernatants. The retroviral supernatant was then added to the wells and the plates were centrifuged (2500 rpm, 90 minutes, 32°C). Cells were incubated overnight at 37°C. On the next day, a second transduction round similar to the first round was performed. Here, 2 mL of supernatant per well was collected from the plates and stored at 37°C. It was re-added after the centrifugation step and supplied with 2 μg/mL protamine sulfate. On day four, the transduction rate was determined by flow cytometry. The cells were counted, washed and resuspended in the appropriate volume of PBS to allow injection of 2x10^6^ transduced T cells in 200 μL PBS per mouse.

### Animal Experiments

Mouse experiments were conducted in accordance with the German Law for the Protection of Animals and were approved by the local authorities (Regierung von Oberbayern). Rag2^-/-^IL-2Rgc^-/-^ recipient mice and CD45.1^+^ C57BL/6J donor mice were bred and kept in-house in specific pathogen-free animal facilities. AAV serotype 2 containing the genome of HBV genotype D was packed with an AAV serotype 8 capsid, as previously reported (26). In both mouse experiments shown in this paper (**Figures 3**–**6**) male and female mice were used. Based on the previous observation that HBV replicates better in AAV-HBV-infected male mice, the AAV-titer was adjusted accordingly: male mice were infected with 1x10^10^ and female mice with 3x10^10^ viral genomes, each in 100 μL PBS injected in the tail vein. For T-cell transfer, 2x10^6^ freshly transduced murine T cells were injected in 200 μL PBS intraperitoneally per mouse (Rag2^-/-^IL-2Rgc^-/-^, 14-20 weeks old). Both, the virus stock solution as well as the T cells were kept on ice until shortly before injection. CID (AP20187 from Takara) was prepared according to the manufacturer’s recommendation using the prepared stock solution (lyophilized dimerizer in 100% ethanol), PEG-400 (100%; Sigma-Aldrich) and Tween (2%; Roth). CID was administered via intraperitoneal injection within 30 min after preparation in a dosage of 10 mg/kg and was kept on ice until shortly before injection. Four similar iC9 depletion experiments were performed and two representative experiments are shown (**Figures 3**–**5**).

### Serological Analyses

Peripheral blood was collected into Microvette 500 LH-Gel tubes (Sarstedt) and centrifuged to separate serum (10 minutes, 5000 g, RT). For measurement of ALT levels, serum was diluted 1:4 with PBS and the Reflotron ALT test was used (Roche Diagnostics). Serum HBs- and HBe-antigen levels were determined on an Architect™ platform using the quantitative HBsAg test (6C36-44; cut-off, 0.25 IU/mL) and the HBeAg Reagent Kit with HBeAg Quantitative Calibrators (7P24-01; cut-off, 0.20 PEI U/mL) (Abbott Laboratories). Serum levels of IFN-γ, TNF-α, IL-6 and MCP-1 were detected via the cytometric bead array mouse inflammation kit (BD) according to the manufacturer’s instructions and measured on a CytoFLEX S flow cytometer (Beckman Coulter). Data analysis was done with the Prism 8 software.

### Isolation of Human and Murine PBMCs, Murine Splenocytes and Murine Liver-Associated Lymphocytes

For the isolation of human PBMCs, blood was collected from healthy donors using a syringe containing heparin. The PBMCs were purified by diluting the blood 1:2 with wash medium (RPMI + 1% Pen/Strep) and layering it on top of Biocoll separating solution (Biochrom) for centrifugation (1200 g, 20 minutes at RT without breaks). For the isolation of murine splenocytes, spleens from donor mice were mashed through a 100 μm cell strainer (Falcon), washed and resuspended in 2 mL of ACK lysis buffer (8 g NH_4_Cl, 1 g KHCO_3_, 37 mg Na_2_EDTA, add to 1 l H_2_O, pH 7.2-7.4) and incubated for 2 minutes at RT to lyse the erythrocytes. The reaction was stopped by addition of 48 mL of mTCM, the splenocytes were pelleted (350 g, 5 minutes, 4°C) and resuspended in the appropriate medium and volume. For isolation of mouse PBMCs, 15 μL of heparinized whole blood from Microvette 500 LH-Gel tubes was incubated with 250 μL ACK lysis buffer for 2 minutes at RT. Cells were then pelleted and resuspended with 200 μL of FACS buffer. For isolation of liver-associated lymphocytes (LAL), livers were collected from mice after perfusion with PBS to remove non-liver associated lymphocytes. The livers were mashed through a 100 μm cell strainer and washed. The cell strainer was repeatedly washed with wash medium followed by additional mashing steps. The flow through including the cells was then pelleted (350 g, 5 minutes, 4°C), resuspended in 12.5 mL collagenase medium [4500 U collagenase type 4 (Worthington) in Williams Medium E (Thermo Fisher Scientific) supplemented with 8.75 μL CaCl_2_ (2,5M)] and incubated at 37°C for 20 min with repeated stirring. After centrifugation, liver leukocytes were purified using an 80%/40% Percoll (GE Healthcare) gradient (1400 g, 20 minutes, RT, without brake).

### *Ex Vivo* Antigen Stimulation

Flat-bottom 96-well non-tissue-plates were coated with HBsAg (2.5 μg/mL in PBS; Roche Diagnostics) or anti-CD3 and anti-CD28 antibodies (10 μg/mL in PBS) for two hours at 37°C and blocked for 30 minutes at 37°C with 2% BSA in PBS. After incubation, transduced T cells, freshly isolated splenocytes or LALs were added in appropriate number (2x10^5^ cells/well) to the plate. Cells were incubated for the indicated time at 37°C. For the analysis of intracellular cytokine expression by ICS, Brefeldin A (1 μg/mL; Sigma-Aldrich) was added to cells two hours after start of stimulation and cells were incubated for additional 14 hours at 37°C.

### Immunohistochemistry

Liver pieces were fixed in 4% paraformaldehyde (PFA; Santa Cruz Biotechnology) for 24 hours and then transferred into PBS until paraffin embedding for histological analyses. After antigen retrieval at 100°C for 30 minutes with EDTA, liver sections (2 μm) were stained with rabbit anti-HBcAg (Diagnostic Biosystems, 1:50 dilution) as primary antibody and appropriate horseradish peroxidase-coupled secondary antibodies. Immunohistochemistry was performed using a Leica Bond MAX system (Leica Biosystems). For analysis, tissue slides were scanned using an Aperio AT2 slide scanner (Leica Biosystems). HBcAg-positive hepatocytes were manually counted after defining cut-offs for the localization, intensity and distribution of the signal in 10 random view fields (20x magnification) per tissue and the mean numbers of HBcAg-positive hepatocytes were quantified per mm^2^.

### Statistical Analysis

Statistical analyses were done with the Prism 8 software. Statistical differences were calculated using the Mann-Whitney test. P-values < 0.05 were considered significant.

## Results

### Induction of iC9 in HBV-Specific T Cells Rapidly Stops Their Effector Function *In Vitro*

**Figure 1:**
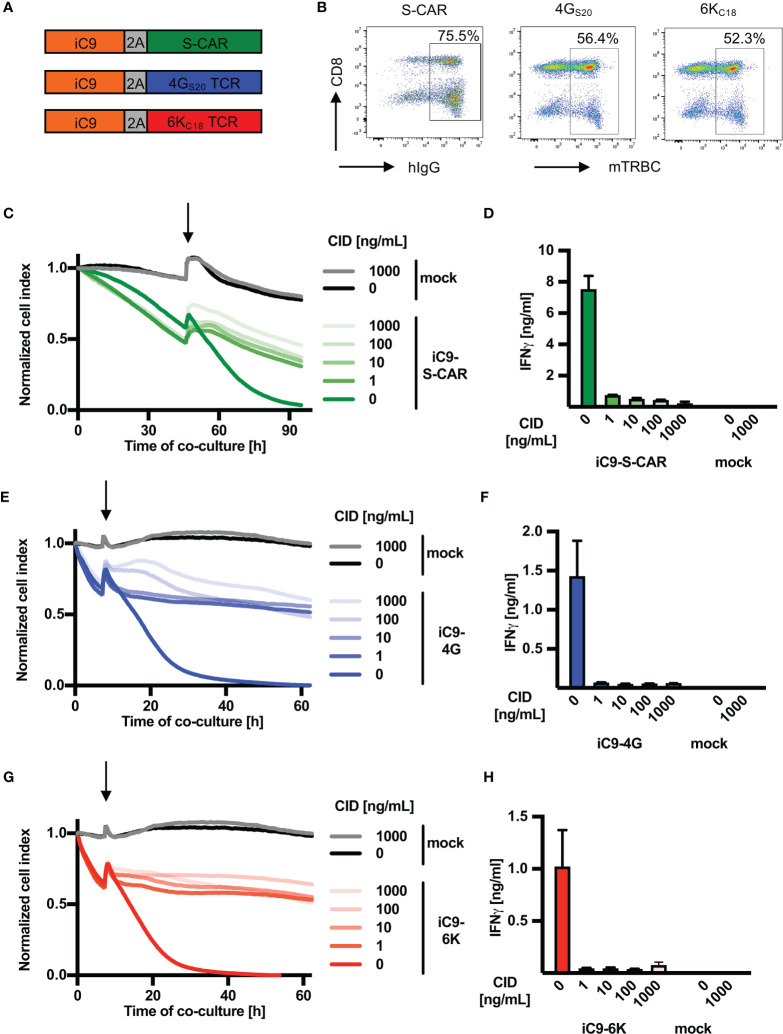
Depletion of HBV-specific T cells via iC9 *in vitro*. **(A)** Schematic representation of the safeguard molecule inducible caspase 9 (iC9) linked to HBV-specific receptors through a T2A element to allow equimolar expression of the proteins. The S-CAR consists of an antibody fragment, which binds to the viral envelope proteins on infected cells, a human IgG1 spacer and CD28/CD3 signaling domains. The TCRs 4G_S20_ (specific for the envelope peptide S20) and 6K_C18_ (specific for the core peptide C18) contain murine constant domains (mTRBC) for better pairing of TCR chains (arranged as beta chain-P2A-alpha chain). **(B)** On day ten after retroviral transduction, cells were stained for hIgG (CAR) or mTRBC (TCRs) and the respective receptor expression was quantified by flow cytometry. **(C–H)** 1.25x10^4^ receptor^+^ T cells co-expressing iC9 were co-cultured with HBV^+^ HepG2.2.15 target cells at an effector to target ratio of 1:4. CID was added (highlighted by an arrow) after the first day in different concentrations ranging from 1 to 1000 ng/mL to activate the inducible caspase 9 cascade leading to T-cell death. **(C, E, G)** Cell viability of target cells was determined with the xCELLigence RTCA in real-time and is displayed as normalized cell index (normalized to the start of co-culture). **(D, F, H)** IFN-γ determined in cell culture medium on day four of the co-culture. This experiment was repeated twice and one representative example is shown. Co-cultures were done in technical triplicates and mean, or mean +/- SEM are shown, respectively.

First, the inducible caspase safeguard system iC9 was cloned into a retroviral vector encoding for HBV-specific receptors. iC9 was linked either to the S-CAR (env-specific), the TCR 4G_S20_ (S-specific) or the TCR 6K_C18_ (core-specific) via a 2A element (**Figure 1A**). All HBV-specific receptors were successfully expressed in 52-75% of T cells after retroviral transduction as quantified by flow cytometry analysis (**Figure 1B**). Co-expression of the safeguard molecule reduced the transduction efficiency of HBV-specific receptors by around 10-40% depending on the receptor and experiment (**Supplementary Figure 1A**). The expression level as determined by the MFI was reduced only in iC9-S-CAR T cells compared to S-CAR T cells not co-expressing iC9. Consequently, S-CAR T-cell killing was slightly delayed, while effector functions of TCR-expressing T cells remained unaltered (**Supplementary Figures 1B–G**). Next, we investigated the effect of inducing the iC9 safeguard mechanism in T cells. To this end, the transduced T cells were co-cultured with HBV^+^ HepG2.2.15 cells and the viability of the HBV-replicating target cell line was measured using a real-time cytotoxicity assay. When around 40% of HBV^+^ target cells were killed, CID was added in different concentrations to induce the iC9 safeguard system. Killing by iC9-S-CAR T cells was reduced within one hour (**Supplementary Figure 2A**), even when the lowest concentration of CID (1 ng/mL) was used (**Figure 1C**). In addition, the HBV-specific production of IFN-γ measured 96 hours later, was reduced by 10-fold (**Figure 1D**). Likewise, cytotoxicity and cytokine production of T cells expressing HBV-specific TCRs were rapidly stopped (**Figures 1E–H** and **Supplementary Figures 2B, C**). Interestingly, compared to TCR-grafted T cells (**Figures 1E, G**) killing by S-CAR T cells was slower (**Figure 1C**). This led to an overall longer duration of the co-culture with S-CAR or mock T cells and probably caused a generally reduced target cell viability after 90 hours (**Figure 1C**). Taken together, induction of the iC9 safeguard mechanism rapidly halted effector functions of HBV-specific T cells.

### Ganciclovir Treatment of HBV-Specific T Cells Expressing the HSV Thymidine Kinase Abrogates their Effector Function *In Vitro*

**Figure 2:**
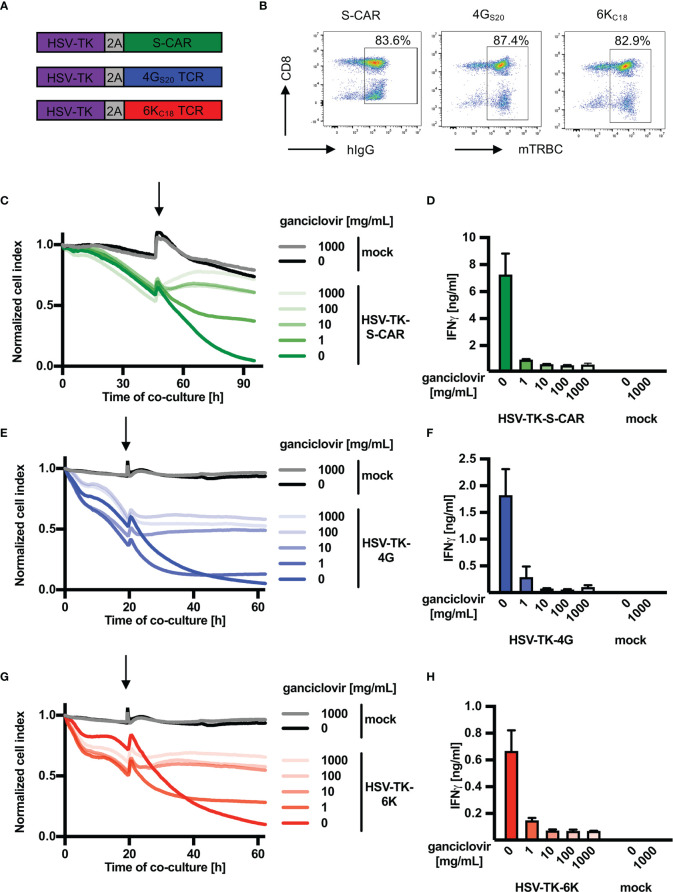
Depletion of HBV-specific T cells via HSV-TK *in vitro*. **(A)** Schematic representation of the safeguard molecule herpes simplex virus thymidine kinase (HSV-TK) linked to either S-CAR or TCR 4G_S20_ or 6K_C18_ through a T2A element. **(B)** Receptor expression of transduced T cells assessed by flow cytometry on day ten after retroviral transduction. **(C–H)** 1.25x10^4^ receptor^+^ T cells co-expressing HSV-TK were co-cultured with HBV^+^ HepG2.2.15 target cells at an effector to target ratio of 1:4. Ganciclovir was added (highlighted by an arrow) after the first day in different concentrations ranging from 1 to 1000 mg/mL to interrupt DNA synthesis and inducing T-cell death **(C, E, G)** Killing of target cells was measured using the xCELLigence real-time cell analyzer and is reported as the normalized cell index relative to the starting point of the co-culture. **(D, F, H)** IFN-γ determined in cell culture medium on day four of the co-culture. This experiment was repeated twice and one representative example is shown. Co-cultures were done in technical triplicates and mean, or mean +/- SEM are shown, respectively.

In analogy to iC9, the safety switch HSV-TK was first cloned into the vector encoding HBV-specific receptors (**Figure 2A**). After retroviral transduction, the rate of T cells expressing an HBV-specific receptor was around 80% for the three constructs (**Figure 2B**). In contrast to iC9, co-expression of HSV-TK in T cells did not alter expression rates of S-CAR or TCRs (**Supplementary Figure 3A**). Nevertheless, HSV-TK co-expression delayed T-cell killing by ten hours (**Supplementary Figures 3B, D, F**) while cytokine production was only slightly reduced in T cells expressing the S-specific TCR 4G_S20_ (**Supplementary Figures 3C, E, G**). Next, HSV-TK T cells were co-cultured with HBV^+^ target cells and GCV was added to induce effector cell death. A titration of GCV was performed including the manufacturer’s recommendation of 10 mg/mL. Depletion of HBV-specific HSV-TK^+^ T cells after administration of ≥ 10 mg/mL GCV halted cytotoxicity (**Figures 2C, E, G**) within less than one hour (**Supplementary Figures 4A–C**) and stopped production of IFN-γ (**Figures 2D, F, H**). Although a concentration of 1 mg/mL GCV was sufficient to stop T-cell effector functions, it was not as efficient as higher concentrations of GCV and occurred only ten hours later (**Figures 2C, E, G**). Overall, we concluded that HSV-TK worked as a very potent safeguard system to stop the effector function of HBV-specific T cells *in vitro.*

### *In Vivo* Induction of iC9 Prevents Toxicity of S-CAR T Cells

Based on the *in vitro* results, both safeguard systems tested seemed equally suitable for depletion of HBV-specific T cells. However, in our hands application of GCV was more difficult as it needs to be kept at very alkaline conditions (approximately pH11) to prevent precipitation. In a first *in vivo* experiment (data not shown), GCV induced tissue irritation at the injection site causing distress for the animals and thereby rendered GCV delivery unreliable.

**Figure 3:**
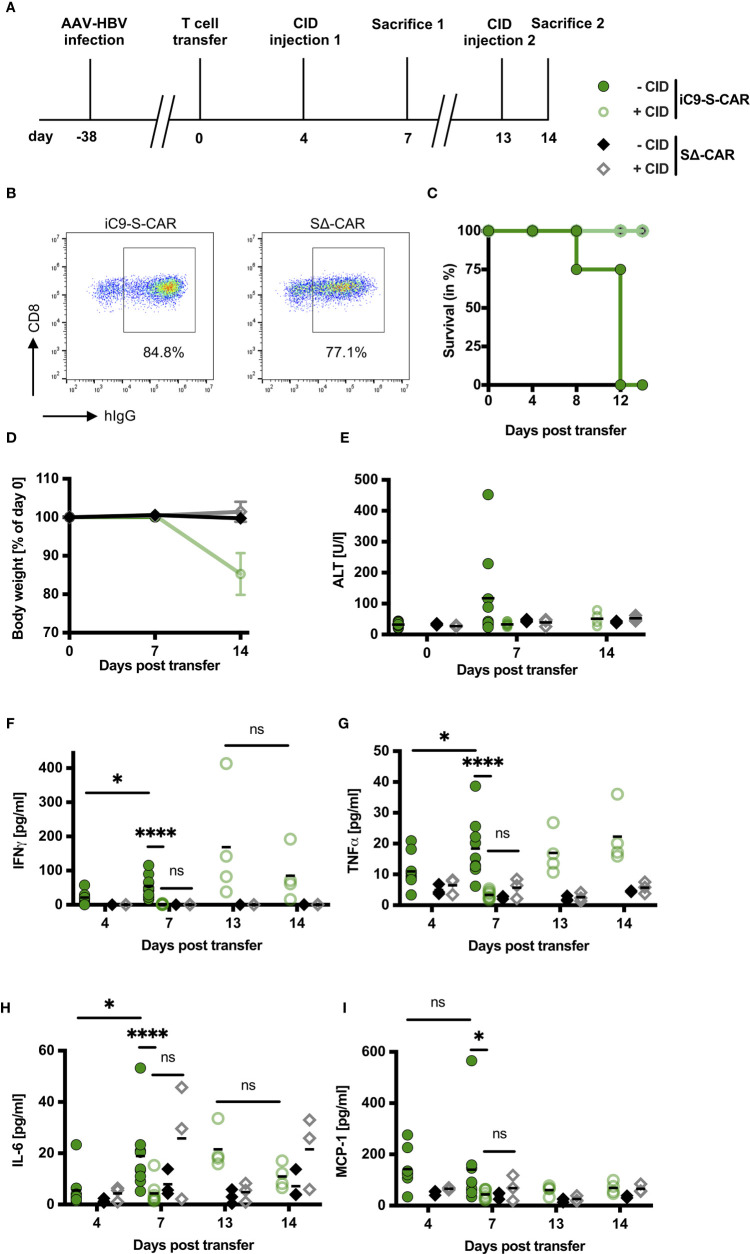
Impact of iC9 induction on side effects of adoptive T-cell transfer *in vivo.* **(A)** Scheme of the experimental procedure. Rag2^-/-^IL-2Rgc^-/-^ mice were infected with HBV using an adeno-associated vector. 1x10^10^ viral genomes for male and 3x10^10^ viral genomes for female mice were injected intravenously per mouse. After establishment of a stable infection around five weeks later, 2x10^6^ iC9-S-CAR T cells (n = 17) or SΔ-CAR T cells (n = 6) were administered i.p. per mouse. The SΔ-CAR, containing the same extracellular domains to bind HBsAg but exchanged intracellular domains rendering it incapable of activating T cells, served as a control for the antiviral effect of T cells and unspecific effects of CID injection. CID or a negative control (preparation identical to CID but without the active substance) was injected i.p. on day four and day 13. Nine mice of the iC9-S-CAR group were sacrificed on day seven, the rest of the mice were sacrificed on day 14. **(B)** Receptor expression of retrovirally transduced T cells assessed by flow cytometry on the day of adoptive T-cell transfer. **(C)** Survival analysis of the mice between transfer of the T cells and sacrifice. **(D)** Change in body weight of the different groups. Individual weights for each mouse on day zero were set to 100%. **(E)** ALT activity measured in mouse sera on day zero, seven and 14 after T-cell transfer. **(F–I)** Cytokines in mouse sera on day four, seven, 13 and 14 after T-cell transfer were determined by cytometric bead array and flow cytometry. Blood on day four and 13 was collected just before CID injection. **(D–I)** Data points represent individual animals and mean values are indicated. Mann-Whitney test: ns, not significant, *p < 0.05, ****p < 0.0001.

Hence, we selected iC9 as the more promising and convenient safeguard system for further evaluation *in vivo*. More specifically, we aimed to adapt our model of chronic hepatitis B (27) to resemble a clinical scenario that might require on-demand depletion of adoptively transferred T cells. To this end, a high titer HBV infection was established in Rag2^-/-^IL-2Rgc^-/-^ mice, resulting in high amounts of circulating HBsAg and infected hepatocytes. The lack of T, B, and NK cells provided “space” for the high number of adoptively transferred T cells to engraft and expand most efficiently. Murine CD45.1^+^ CD8^+^ T cells were engineered to co-express iC9 and the S-CAR since it functions independently of human MHC molecules and is hence easier to apply. CID was administered on day four and 13 post T-cell transfer and one group of mice was sacrificed on day seven, when hepatotoxicity was expected to peak (6, 27) and one group on day 14 (**Figure 3A**). Cells expressing a decoy-CAR (SΔ-CAR) that can bind HBsAg but does not carry signaling domains served as a control for unspecific effects of CID towards T-cell survival or HBV replication (**Figure 3B**). All mice of the group that had received S-CAR T cells, but no CID, died between day eight and day twelve, while those mice, in which S-CAR T cells were depleted, survived (**Figure 3C**). The death of the animals was not preceded by a loss in body weight (**Figure 3D**) and ALT levels had only slightly increased to on average of 150 U/l (**Figure 3E**), indicating that rather a general and not a liver-directed immune reaction caused the fatalities. Mice, in which S-CAR T cells were depleted, lost around 15% of their body weight by day 14 (**Figure 3D**) and induction of the iC9 safeguard mechanism prevented the moderate hepatotoxicity (**Figure 3E**).

To evaluate a potential cytokine release syndrome (28), we measured the serum concentration of inflammatory cytokines over the course of the experiment. The cytokines IFN-γ, TNF-α, IL-6 and MCP-1 were slightly elevated four days post transfer of HBV-specific T cells but not of control T cells (**Figures 3F–I**) and IFN-γ, TNF-α and IL-6 further increased significantly in the mice without T-cell depletion (**Figures 3F–H**). However, three days after injection of CID all cytokines had returned to background levels (**Figures 3F–I**). Interestingly, this effect was only temporary and cytokine levels rose again one week after T-cell depletion. A second injection of CID reduced IFN-γ and IL-6 levels within one day (**Figures 3F, H**) but not TNF-α and MCP-1 (**Figures 3G, I**). Taken together, induction of the iC9 safeguard system in HBV-specific T cells efficiently prevented liver damage and stopped systemic presence of inflammatory cytokines. This pointed to an efficient depletion of iC9-S-CAR T cells.

### *In Vivo* Induction of iC9 Reduces Numbers of Transferred T Cells and Renders Them Non-Functional

**Figure 4:**
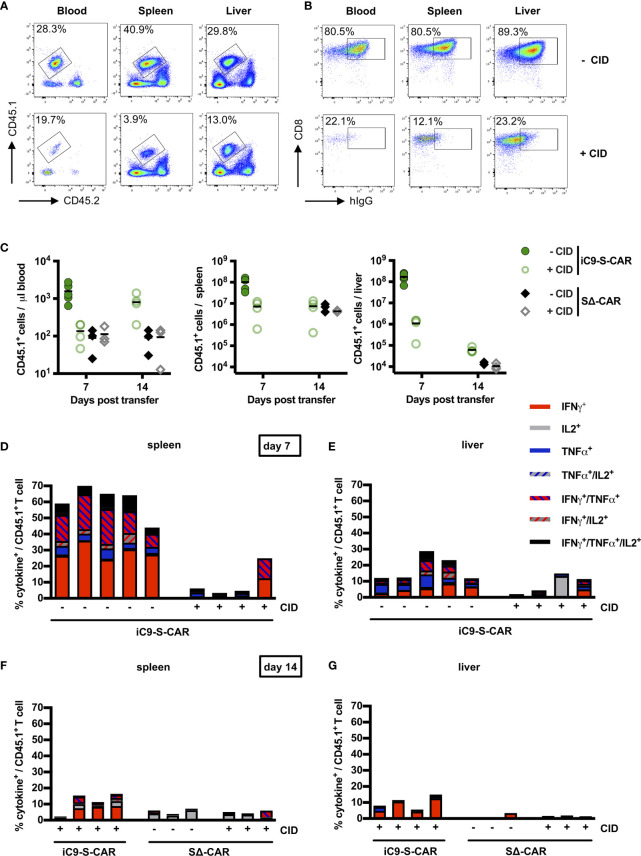
Depletion of transduced T cells via iC9 *in vivo.* **(A)** Exemplary flow cytometry plot of transferred CD45.1^+^ T cells found in peripheral blood, in the spleen and in the liver. Both, a mouse which did not receive (upper row) and a mouse which received CID (lower row), were sacrificed on day seven post T-cell transfer. The congenic marker CD45.1 allowed to differentiate transferred cells from the endogenous CD45.2^+^ cells of the recipient mice. **(B)** Exemplary flow cytometry plot of CD8^+^ and CAR T cells in peripheral blood, in the spleen and in the liver of the same mice as shown in **(A)**. **(C)** Count of transferred CD45.1^+^ T cells in the peripheral blood, the spleen and the liver on day seven and day 14 after T-cell transfer. Absolute count of cells was determined by flow cytometry using Counting Beads. The result was extrapolated to the concentration in blood or to the whole organ considering the proportion that was used to isolate splenocytes or LALs. Data for the iC9-S-CAR group on day 14 are not available because the animals died between day seven and 14. **(D–G)** *Ex-vivo* functionality of transferred CD45.1^+^ T cells collected from spleen (**D** day seven, **F** day 14) or liver (**E** day seven, **G** day 14). Intracellular cytokine expression (TNF-α, IFN-γ and IL-2) was determined after overnight-culture on plate-bound HBsAg. **(C)** Data points represent individual animals and mean values are indicated. **(D–G)** Each column represents an individual animal.

In order to quantify the depletion of transferred T cells in blood, but also spleen and liver, mice were sacrificed on day seven and 14. The congenic marker CD45.1/2 allowed for easy differentiation of transferred CD45.1^+^ cells from endogenous CD45.2^+^ immune cells (**Figure 4A**). Although CD45.1^+^ cells were not completely depleted after CID injection (**Figure 4A**, lower row), additional staining for the S-CAR revealed that especially T cells with a high S-CAR expression were targeted and had completely vanished from all examined compartments (**Figure 4B**). On day seven, numbers of transferred T cells in the blood and the spleen were reduced by 1.5log_10_ and in the liver by 2-3log_10_ in comparison to mice in which suicide of HBV-specific T cells had not been induced (**Figure 4C**). The number of transferred cells that remained after depletion was comparable to the number of SΔ-CAR T cells, which did not get a specific stimulus for proliferation. A second CID administration on day 13, with the idea to eliminate the remaining cells that had not been depleted by the first CID administration, was most effective in the liver but did not lead to the complete elimination of cells (**Figure 4C**).

In addition, the isolated T cells were assessed for their functionality. To this end, LALs and splenocytes were incubated on HBsAg-coated plates and stained for secretion of IFN-γ, TNF-α and IL-2 (**Supplementary Figure 5A**). Sixty percent of CD45.1^+^ T cells retrieved from the spleen, i.e. most of the S-CAR T cells, exhibited HBsAg-specific production of cytokines, with IFN-γ being most prominent (**Figure 4D**). This expression was almost completely abrogated in three out of four mice that had received CID and in line with the observation that mostly S-CAR^hi^ T cells had been depleted (**Figure 4B**). Nevertheless, the CD45.1^+^ T cells could still be activated non-specifically by anti-CD3/anti-CD28 stimulation, further indicating that only the transduced HBV-specific T cells had been depleted (**Supplementary Figure 5**). Interestingly, in this experiment the functional profile of LALs differed from that of splenocytes. Despite a high expression of the S-CAR in the group without depletion (**Figure 4B**), only an average of 15% secreted cytokines upon restimulation with HBsAg (**Figure 4E**). Injection of CID successfully depleted HBV-specific cells in half of the mice. Despite a second injection of CID, few HBV-specific T cells remained detectable in spleen and liver (**Figures 4F, G**). Taken together, depletion of iC9-S-CAR T cells was very efficient, albeit not complete, and their functionality was diminished.

### Specific Depletion of S-CAR T Cells Reduces the Antiviral Effect of the Adoptive T-Cell Transfer

**Figure 5:**
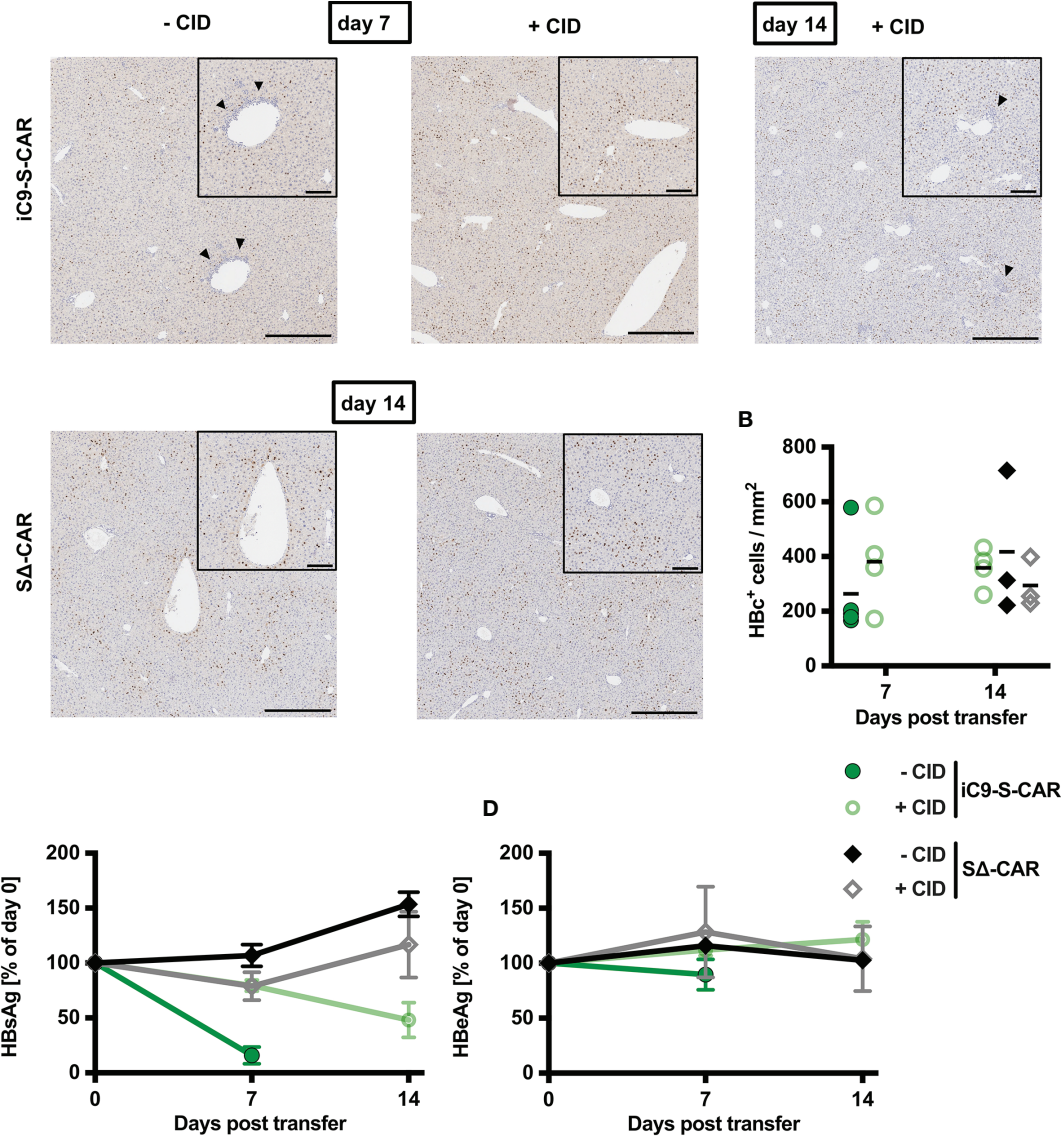
Antiviral effect of iC9-S-CAR T cells *in vivo.* **(A)** Exemplary (1 mouse per group) liver immunohistochemistry stainings for HBcAg-positive hepatocytes. Bars represent 200 µm in the overview and 80 µm in the inlay of the central vein. Arrows indicate lymphocyte infiltrates. **(B)** Quantification of histological analysis of HBcAg expression including all the mice from all the different groups. **(C, D)** HBsAg and HBeAg levels in the serum over time relative to day zero determined by diagnostic assays. **(B)** Data points represent individual animals and mean values are indicated. **(C, D)** Data are given as mean values +/- SD.

Given the successful depletion of HBV-specific T cells (**Figure 4**) accompanied by a lack of cytotoxic T-cell activity in the liver and cytokine secretion (**Figure 3**), we set out to quantify how much this safety measure would come at the cost of the therapeutic, antiviral efficacy of adoptive T-cell transfer. Histological analysis of liver tissue revealed that upon CID injection immune cells did not accumulate near the central veins anymore (**Figure 5A**) and concomitantly the number of HBcAg^+^ hepatocytes remained as high as in control groups (**Figures 5A, B**). Similarly, the effect of S-CAR T cells on levels of circulating HBsAg was completely abrogated three days after the first CID injection (**Figure 5C**). In contrast to that, the second CID injection that had also spared some remaining HBV-specific T cells (**Figures 4F, G**), still allowed S-CAR T cells to reduce HBsAg by 50% between day 7 and day 14 (**Figure 5C**). Levels of HBeAg remained unaltered throughout the treatment in all groups (**Figure 5D**). Taken together, delayed reduction of HBsAg and steady levels of HBcAg suggested that S-CAR T-cell depletion hampered not only the side effects of T-cell therapy but also their antiviral effect.

### Suicide of HBV-Specific T Cells Can Be Induced Within One Hour *In Vivo*

**Figure 6:**
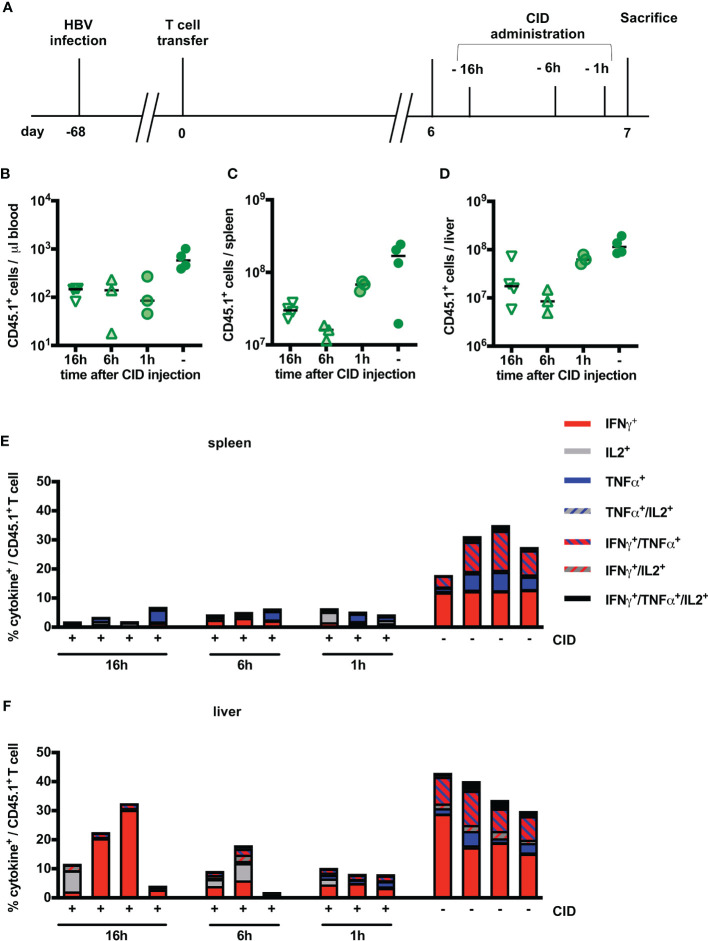
Kinetics of iC9-mediated T-cell depletion *in vivo.* **(A)** Scheme of the experimental procedure. Infection of Rag2^-/-^IL-2Rgc^-/-^ mice with HBV was achieved using an adeno-associated vector. Similar to **Figure 3**, 1x10^10^ viral genomes for male and 3x10^10^ viral genomes for female mice were injected intravenously per mouse. After establishment of a stable infection around 5 weeks later, 2x10^6^ iC9-S-CAR T cells (n = 14) were administered i.p. per mouse. CID was administered between day six and seven and mice were sacrificed on the same day but at different timepoints after CID administration 1h (n = 3), 6h (n = 3) and 16h (n = 4). 4 additional mice received no CID. **(B–D)** Count of transferred CD45.1^+^ T cells in the peripheral blood **(B)**, the spleen **(C)** and the liver **(D)** on different timepoints after CID administration. Count of transferred CD45.1^+^ T cells in the peripheral blood, the spleen and the liver on day seven and day 14 after T-cell transfer. Absolute count of cells was determined by flow cytometry using Counting Beads. The result was extrapolated to the concentration in blood or to the whole organ considering the proportion that was used to isolate splenocytes or LALs. **(E, F)** *Ex vivo* functionality of transferred CD45.1^+^ T cells in the spleen **(E)** and the liver **(F)** via measurement of intracellular cytokine expression (TNF-α, IFN-γ and IL-2) determined after overnight-culture on plate-bound HBsAg. **(B–D)** Data points represent individual animals and mean values +/- SD are indicated. **(E, F)** Each column represents an individual animal.

From our *in vitro* experiments, we had learned that induction of the iC9 safeguard immediately prevented further cytotoxicity of HBV-specific T cells. We next asked, whether this would also hold true *in vivo*. Six days post transfer of iC9-S-CAR T cells, CID was injected 16, six and one hour before analyzing blood, spleen and liver of the mice (**Figure 6A**). The number of transferred T cells in blood was reduced by around 1 log_10_ already one hour after induction of the safeguard system (**Figure 6B**). In contrast to that, in spleen and liver the reduction of transferred T cells was more profound six and 16 hours post CID injection (**Figures 6C, D**). After one hour, the depletion rate was only around 50% in both organs compared to the cell numbers at earlier time points of depletion. Regardless of the time point, HBV-specific T cells in the spleen were efficiently depleted from around 30% to 5% of cytokine secreting cells (**Figure 6E**). In the liver, the rate of HBV-reactive T cells was reduced from around 40% to 10% already one hour after induction of T-cell suicide (**Figure 6F**). Surprisingly, the number of HBV-specific T cells in the liver was higher when CID had been injected 16 hours, before sacrifice compared to an exposure time of six or one hour. In summary, the velocity of T-cell suicide via iC9 dimerization was high and comparable *in vitro* and *in vivo*.

## Discussion

Adoptive T-cell therapy for the treatment of chronic hepatitis B aims to overcome the scarce and narrow T-cell response (3, 29, 30) by applying highly potent, genetically engineered HBV-specific T cells that are able to clear HBV infection via cytotoxicity and cytokine secretion (5, 8). In this study, we evaluated iC9 and HSV-TK as safeguard systems in the context of adoptive T-cell transfer for treatment of persistent HBV infection. *In vitro*, induction of iC9 or HSV-TK co-expressed in HBV-specific-TCR and S-CAR T cells immediately halted T-cell cytotoxicity and cytokine production. *In vivo*, induction of iC9 in S-CAR T cells led to a strong and fast depletion of transferred T cells and prevented liver toxicity and cytokine release. This, however, came at the costs of a loss of antiviral efficacy.

The concept of equipping cells with a safeguard before adoptive transfer originates from stem cell transfer to counteract side effects such as graft-versus-host disease (31). HSV-TK (19, 32), iC9 (33, 34) and a truncated form of the epidermal growth factor receptor (EGFR, targeted by the antibody Cetuximab) (35) are the most clinically advanced and especially iC9 and tEGFR have since been adapted in adoptive T- cell therapy to deplete CAR- or TCR-redirected T cells on demand (36, 37). Given that EGFR has been reported to be expressed on regenerative clusters of hepatocytes (38) and that we were seeking a safeguard that prevents excessive liver damage, we did not select tEGFR but focused on HSV-TK and iC9.

So far, suicide switches have been mostly applied for CAR T cells (36, 37) and the usage for iC9 has only been described for one TCR (39). In our study, co-expression of either suicide switch *per se* did not alter the expression or functionality of HBV-specific T cells considerably. This was also true for the TCR, which as a heterodimer might be more sensitive to additional genes being expressed from the same transgene. Both, induction of HSV-TK and iC9, was equally fast and effective in stopping HBV-specific T cells from executing their designated effector functions. Others have characterized HSV-TK as being as potent as iC9 but rather slow in inducing T-cell death *in vitro* (22) and requiring multiple GCV injections in patients (40). A reason why HSV-TK induction was faster in our system could be that T cells were in a highly active state when treated with GCV and were potentially more prone to DNA incorporation of GCV. Nevertheless, we decided against a further *in vivo* evaluation of the HSV-TK suicide switch because of its reported immunogenicity (41), the side and bystander effects of GCV (21, 42, 43), and the technical difficulty of injecting the alkaline GCV solution into the thin murine veins (43).

The iC9 system provides an interesting alternative because it is poorly immunogenic as it only contains human-derived domains, the dimerizer for its induction is biologically inert and was proven to be safe in healthy volunteers (44). The mechanism of dimerizer activation is independent of the cell cycle and iC9 was shown to be highly effective and very fast in both preclinical and clinical studies (18, 45), which prompted us to evaluate iC9 also in our *in vivo* model.

We have previously shown that application of S-CAR T cells in HBV-replicating mice is safe. In immune competent HBV transgenic mice (6) and tolerized AAV-HBV infected mice (27), and likewise in the present study, the grafted T cells only induced a moderate liver damage while slowly reducing viral loads. Here, in order to simplify testing of the iC9 safety switch, we adjusted the model to allow exuberant S-CAR T-cell activation risking non-liver specific side effects. To this end, the S-CAR used in this study was codon-optimized and carried the natural IgG1-spacer, leading to a higher expression and most powerful activation (data not shown) including potential off-target activation via binding to Fc-receptor bearing cells, as opposed to the non-codon-optimized S-CAR with an immune-silenced IgG1-spacer (46) used previously (27). Furthermore, a high dosage of 2x10^6^ S-CAR-CD8^+^ T cells (equivalent to approx. 6x10^9^ per 75 kg patient) was injected into immune incompetent mice allowing rapid T cell expansion. These modifications of the model resulted in 20-fold higher T cell numbers in the blood and likely facilitated a strong, systemic T cell activation, which was unforeseen and unfortunately let to the fatalities observed in this study. These high numbers of engrafted T cells allowed for better quantification of T cell depletion on a logarithmic scale, which was not feasible when we aimed at quantifying T cell depletion in above mentioned immune competent mouse models (data not shown). For the assessment of side effects of T-cell therapies, preclinical *in vivo* models should mimic the clinical setting as precisely as possible. By using a mouse model lacking T, B and NK cells we cannot fully investigate the interactions of the transferred T cells with the endogenous immune cells and how this might possibly impact on the safety of T-cell therapy. In fact, although CAR T cells are known to initate a possible CRS, the endogenous immune system plays a key role in its clinical manifestation (47).

In our set-up, we were able to rescue mice from excessive S-CAR T-cell activation by a single CID injection preventing moderate hepatotoxicity and halting cytokine release. One CID administration led to a reduction of transferred T cells by over 90% in blood and spleen and over 99% in the liver. This is in line with the depletion rates in blood that other studies in mice (48–51), macaques (52) and men (53–55) have reported. In fact, considering that most of the remaining transferred cells had no or at best a low expression of the S-CAR and showed low *ex vivo* reactivity towards HBsAg, the specific depletion of iC9-S-CAR T cells was presumably even higher than 99% in our setting.

Nevertheless, transferred T cells were not completely eliminated. The escape of T cells with low receptor expression or little activation even from repeated CID (55) has been observed in other models and several reasons have been discussed. It has been proposed, for example, that activation positively influences transgene expression and T cells were shown to be susceptible again to CID when they had been stimulated *ex vivo* (51, 55). This would explain why depletion after CID was in our model most effective in the liver, the site where S-CAR T cells encountered their cellular target. Furthermore, in the setting of hematopoietic stem cell transplantation with iC9-expressing T cells, virus-specific T cells recovered more easily than alloreactive T cells (54). It was suggested that they are more resistant to apoptotic signals (54) and the addition of antiapoptotic agents could increase sensitivity towards CID (56). Indeed, it was reported that cells that are resistant to CID have upregulated levels of B-cell lymphoma 2 (Bcl-2) protein, which as a target of caspase 3 could prevent a lower threshold for apoptosis (52). Interestingly, HBV-specific T cells have been shown to differ in their Bcl-2 levels depending on their antigen-specificity, with polymerase-specific T cells expressing significantly less Bcl-2 than core-specific T cells (57). Assuming that this phenomenon was dependent on the site of antigen encounter, i.e. presumably the liver for polymerase-specific T cells and lymphoid organs for core-specific T cells due to circulating HBeAg, this could explain why iC9-S-CAR T cells were particularly susceptible towards CID. Intriguingly, a second CID on day 13 seemed to spare iC9-S-CAR T cells in blood and spleen but not in the liver, supporting the notion that the liver environment induced a special T-cell phenotype.

Most studies so far have been performed in xenograft models or in humans, relying only on blood analyses. By contrast, our model is a syngeneic one using murine T cells that can interact with murine tissue and might be the reason for the particular depletion kinetics observed. In human studies, many of the iC9-expressing T cells were depleted as fast as 30 minutes after CID (53, 55). In our experiments, depletion of iC9-S-CAR T cells was at its maximum one hour after CID while numbers in spleen and liver were further reduced six hours after CID, which could be a result of the pharmacokinetics of the dimerizer. Interestingly, T-cell numbers were higher in spleen and liver when CID was done 16 hours compared to six hours before sacrifice. We can only speculate that the remaining T cells had started proliferating again, and that the reported short cell cycle durations of activated T cells of around six hours (58) reflected on the retrieved T-cell numbers. There is no doubt that some of the iC9-S-CAR T cells were still active after the first CID on day seven, since we observed reduced levels of HBsAg, loss of body weight and an increase in inflammatory cytokines in this group. This might differ from other studies, in which iC9-CD19-CAR T cells, once depleted, remained steady at low levels (50, 51) maybe due to a lack of stimulus in these xenograft models.

Not surprisingly, depletion of iC9-S-CAR T cells came at the cost of delaying any antiviral activity as HBsAg only started to drop by day 14 and HBcAg^+^ hepatocytes and HBeAg remained unchanged. Using S-CAR T cells to treat AAV-HBV infection, we have previously observed a fast drop of HBsAg and an up to two week delay in HBeAg decrease (27), which can be explained by HBsAg being bound by S-CAR T cells leading to a decrease of free, measurable HBsAg. A lack of tumor cells being controlled after CID has also been observed in lymphoma models (51, 59). This reflects the principal dilemma of adoptive T-cell therapy, in which T cell-related safety and efficacy are inherently linked together.

Apart from the iC9 suicide switch, other strategies to manage acute toxicities can be considered. The IL-6 receptor blocking antibody tocilizumab is very efficient in reverting CRS symptomology (16). However, during CRS, IL-6 is mostly produced by monocytes (60) and blocking its activity will not directly affect T-cell effector function and hence not prevent T-cell-related toxicity. Another therapeutic option is the administration of corticosteroids that have strong immunosuppressive properties. They have been shown to be effective in the management of CRS that occurred after T-cell therapy (16, 47, 61). Nonetheless, the long-term use of systemic corticosteroids has side effects and can lead to a reactivation of HBV infection or enhance the viral replication (62) and would thus diminish the chances of the host´s endogenous HBV-specific immune response to be reinvigorated by adoptive T-cell therapy. *Mestermann et al.* recently proposed an elegant way to switch T-cell functionality on and off on demand by using the tyrosine kinase inhibitor Dasatinib. In their system, Dasatinib rapidly paused complete T-cell functionality while dexamethasone had only a partial effect on cytotoxicity and IFN-γ secretion (63). However, in the end, when adoptively transferred T cells have cleared the infection, it would also be desirable to have a safeguard at hand that targets and eliminates specifically only the genetically modified T cells. In our hands, iC9 seems to be a convenient and reliable safeguard mechanism to do so as proven by its fast and effective T cell depletion and prevention of toxicity in our model of adoptive T-cell therapy of chronic hepatitis B.

## Data Availability Statement

The raw data supporting the conclusions of this article will be made available by the authors, without undue reservation.

## Ethics Statement

The animal study was reviewed and approved by the District Government of Upper Bavaria (permission number: 02-17-227).

## Author Contributions

AK and SS conducted the *in vitro* experiments. AK and ADK performed the *in vivo* experiments. AK, UP, and KW designed the experiments. AK and KW analyzed the data. MP provided essential material. UP provided critical infrastructure. AK and KW wrote the manuscript. All authors contributed to the article and approved the submitted version.

## Funding

The work was funded by the German Research Foundation (DFG) *via* TRR36 as a stipend to AK and by the German Center for Infection Research (DZIF) as a young investigator grant (05.812) to KW.

## Conflict of Interest

KW and UP are co-founders and shareholders of SCG Cell Therapy Pte. Ltd.

KW is partially employed by SCG Cell Therapy GmbH.

The remaining authors declare that the research was conducted in the absence of any commercial or financial relationships that could be construed as a potential conflict of interest.

## Publisher’s Note

All claims expressed in this article are solely those of the authors and do not necessarily represent those of their affiliated organizations, or those of the publisher, the editors and the reviewers. Any product that may be evaluated in this article, or claim that may be made by its manufacturer, is not guaranteed or endorsed by the publisher.
