# Supplementary material for: Depletion of T cells via Inducible Caspase 9 Increases Safety of Adoptive T-Cell Therapy Against Chronic Hepatitis B
Source: Front Immunol. 2021 Oct 6;12:734246. doi: 10.3389/fimmu.2021.734246 (PMC8527178; doi:10.3389/fimmu.2021.734246)
Supplement: Supplementary file 1 [file DataSheet_1.pdf]

Supplementary Material

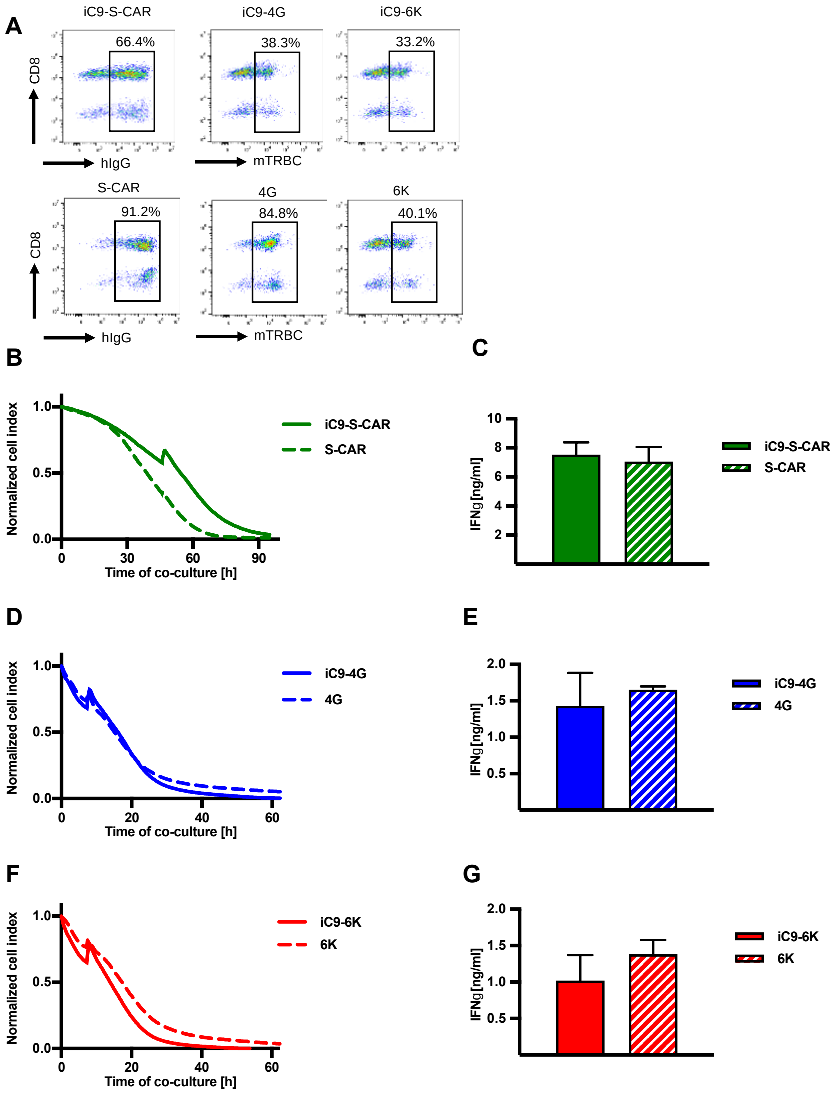

Supplementary Figure 1. ***In vitro*** **comparison of HBV-specific T cell functionality with or without iC9 co-expression. (A)** T cells were simultaneously transduced either with a retroviral vector coding for an HBV-specific receptor alone or with the newly cloned retroviral vectors containing iC9 linked to HBV-specific receptors through a T2A element. On day 10 after retroviral transduction, cells were stained for hIgG (CAR) or mTRBC (TCRs) and the respective receptor expression was assessed by flow cytometry. **(B-G)** 1.25 x 10^4 receptor$^+$ T cells which co-expressed or did not co-express iC9 were co-cultured with HBV$^+$ HepG2.2.15 target cells at an effector to target ratio of 1:4. **(B, D, F)** Cell viability of target cells was determined with the xCELLigence RTCA in realtime and is displayed as normalized cell index (normalized to the start of co-culture). **(C, E, G)** IFN-γ determined in cell culture medium on day four of the co-culture. Co-cultures were done in triplicates and mean, or mean +/- SEM are shown, respectively.

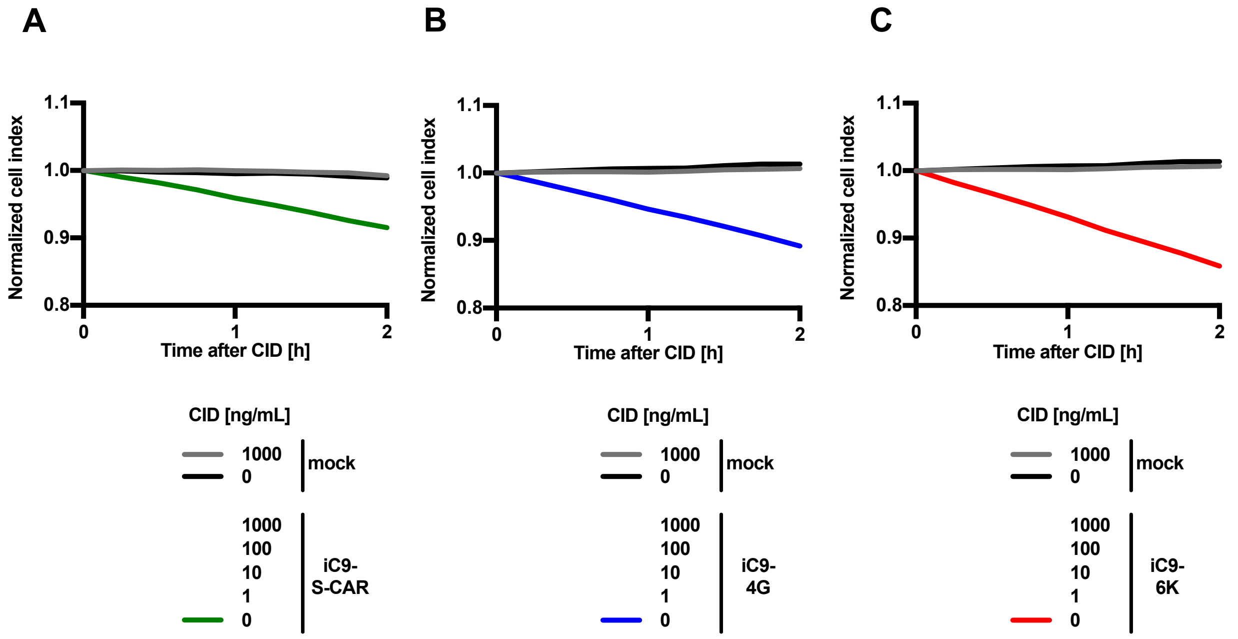

Supplementary Figure 2. Kinetics of iC9-mediated depletion of HBV-specific T cells *in vitro*. (A-C) 1.25x10^4 receptor$^+$ T cells co-expressing iC9 were co-cultured with HBV$^+$ HepG2.2.15 target cells at an effector to target ratio of 1:4. CID was added after the first day in different concentrations ranging from 1 to 1000 mg/mL to activate the inducible caspase 9 cascade leading to T-cell death. **(A, B, C)** Killing of target cells was measured using the xCELLigence real-time cell analyzer and is reported as the normalized cell index relative to the addition of CID. This experiment was repeated twice and one representative example is shown. Co-cultures were done in technical triplicates and mean are shown.

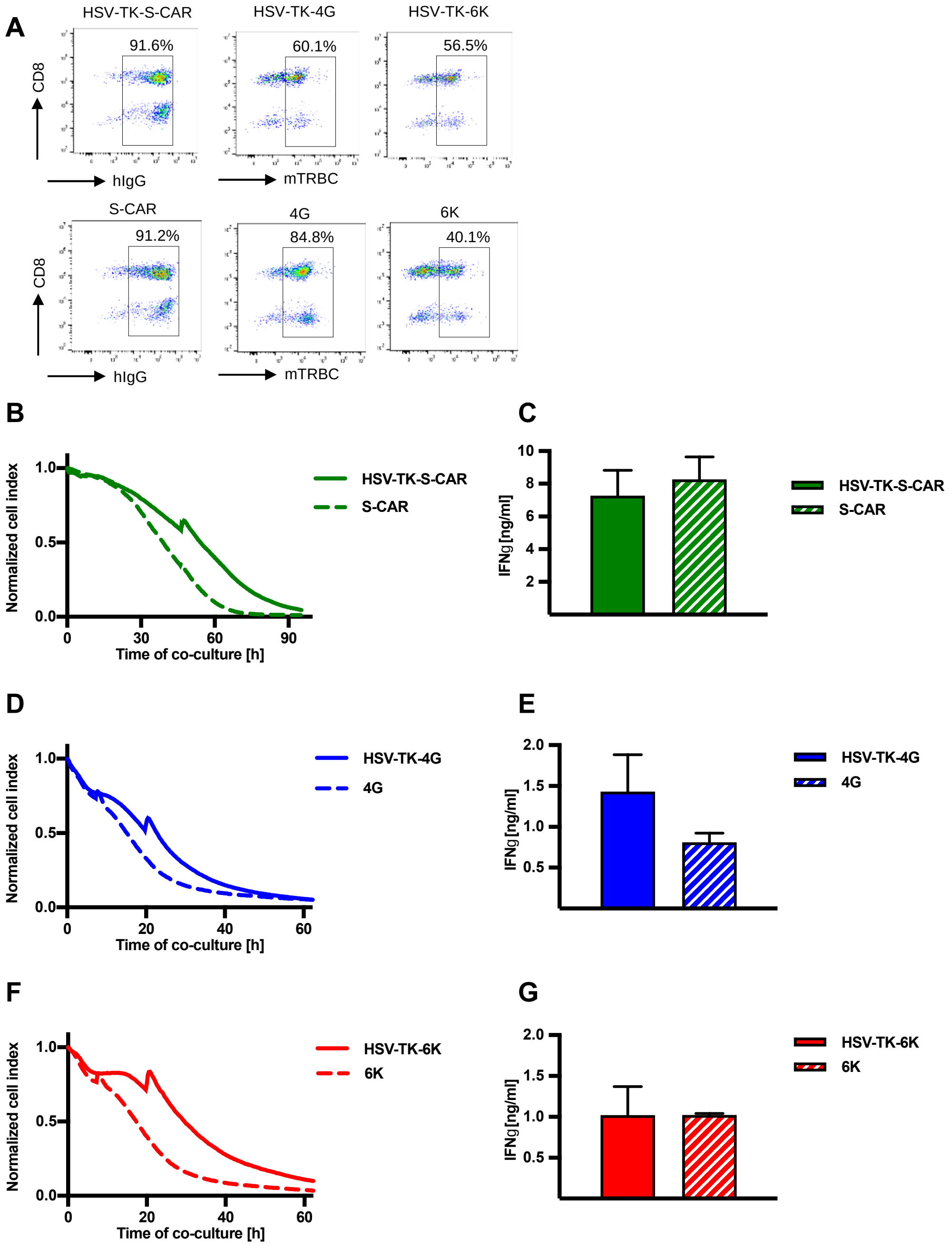

Supplementary Figure 3. ***In vitro*** **comparison of HBV-specific T cell functionality with or without HSV-TK co-expression. (A)** T cells were retrovirally transduced to either co-express an HBV-specific receptor and HSV-TK or to express an HBV-specific receptor alone. Receptor expression of transduced T cells was assessed by flow cytometry on day 10 after retroviral transduction. **(B-G)** 1.25 x 10^4 receptor$^+$ T cells co-expressing or not co-expressing HSV-TK were co-cultured with HBV$^+$ HepG2.2.15 target cells at an effector to target ratio of 1:4. **(B, D, F)** Killing of target cells was measured using the xCELLigence real-time cell analyzer and is reported as the normalized cell index relative to the starting point of the co-culture. **(C, E, G)** IFN-γ determined in cell culture medium on day four of the co-culture. Co-cultures were done in triplicates and mean, or mean +/- SEM are shown, respectively.

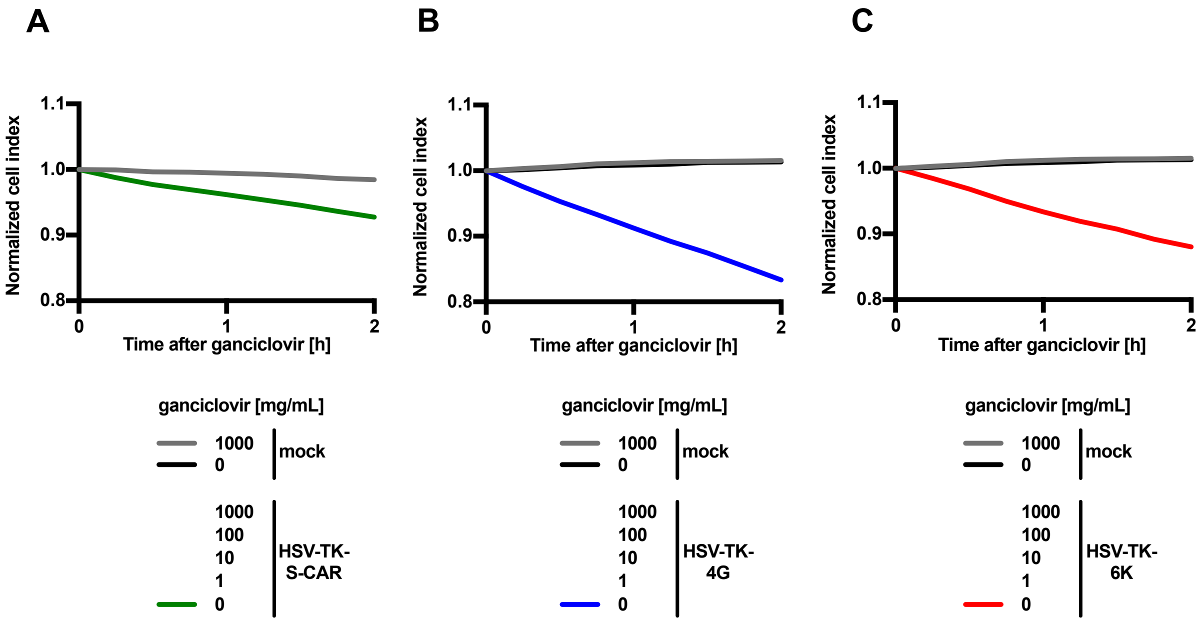

Supplementary Figure 4. Kinetics of HSV-TK-mediated depletion of HBV-specific T cells *in vitro*. (A-C) $1.25x10^4$ $receptor^+$ T cells co-expressing HSV-TK were co-cultured with HBV^+ HepG2.2.15 target cells at an effector to target ratio of 1:4. Ganciclovir was added after the first day in different concentrations ranging from 1 to 1000 mg/mL to interrupt DNA synthesis and inducing T-cell death **(A, B, C)** Killing of target cells was measured using the xCELLigence real-time cell analyzer and is reported as the normalized cell index relative to the addition of ganciclovir. This experiment was repeated twice and one representative example is shown. Co-cultures were done in technical triplicates and mean are shown.

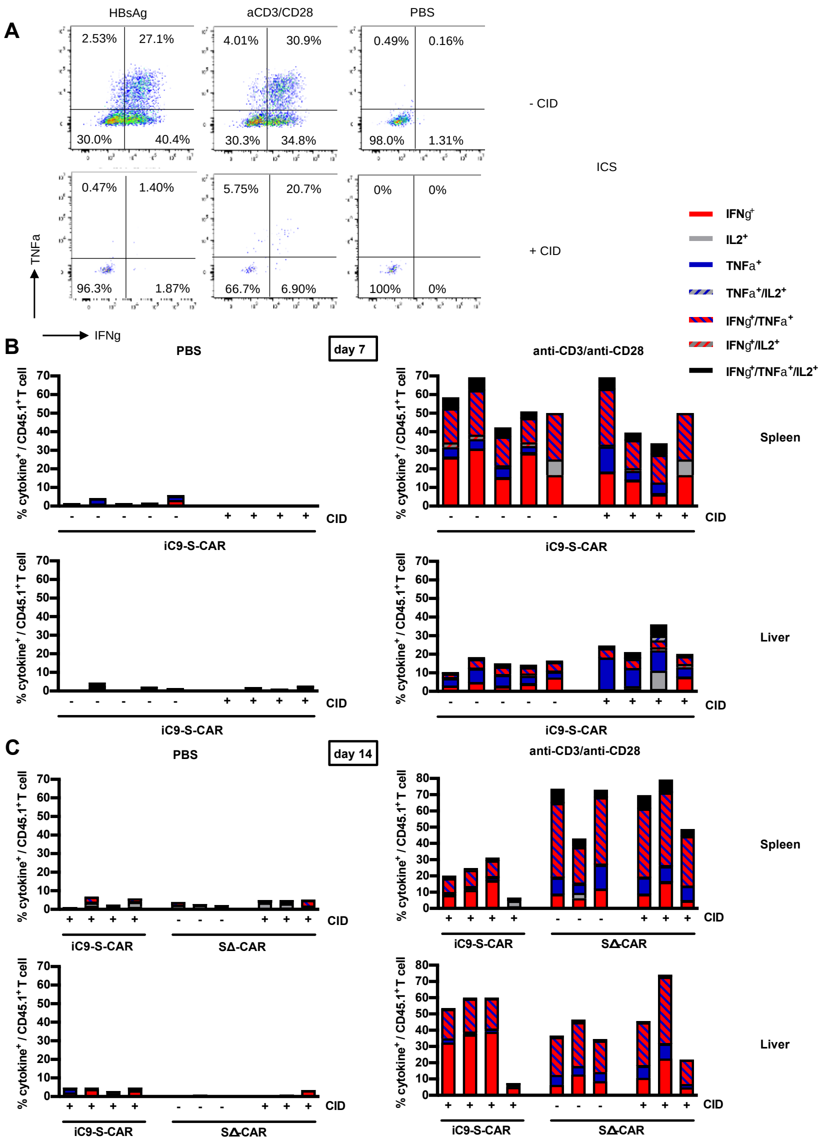

Supplementary Figure 5. Intracellular cytokine staining of adoptively transferred HBV-specific T cells. Rag2$^{-/-}$IL-2Rgc$^{-/-}$ mice were infected with HBV using an adeno-associated vector. After establishment of a stable infection around 5 weeks later, 2×10^6 iC9$^+$-S-CAR$^+$ T cells (n = 17) or unfunctional SΔ-CAR$^+$ T cells (n = 6) were administered i.p. per mouse. CID or a negative control (similar preparation than CID but without the active substance) was injected i.p. on day 4 and day 13. Nine mice of the iC9$^+$-S-CAR$^+$ group were sacrificed on day seven, the rest of the mice were sacrificed on day 14. **(A)** Exemplary flow cytometry plots and gating strategy of spleen-derived T cells cultured overnight on HBsAg-, anti-CD3/anti-CD28- or PBS-coated control plates are shown. Upper row: mouse which did not receive CID, lower row: mouse which did receive CID. Both mice were sacrificed on day seven. Expression of IFN-γ and TNF-α is shown on transferred CD45.1$^+$ T cells. **(B,C)** Ex-vivo functionality of transferred CD45.1$^+$ T cells in the spleen and the liver (B day 7, C day 14) was determined via measurement of intracellular cytokine expression (TNF-α, IFN-γ and IL-2) after overnight-culture on plate-bound HBsAg (Figure 4D-G) for specific restimulation, on plate-bound anti-CD3/anti-CD28 antibodies (right side) for unspecific restimulation and on PBS (left side) as a negative control. (B,C) Each column represents an individual animal.

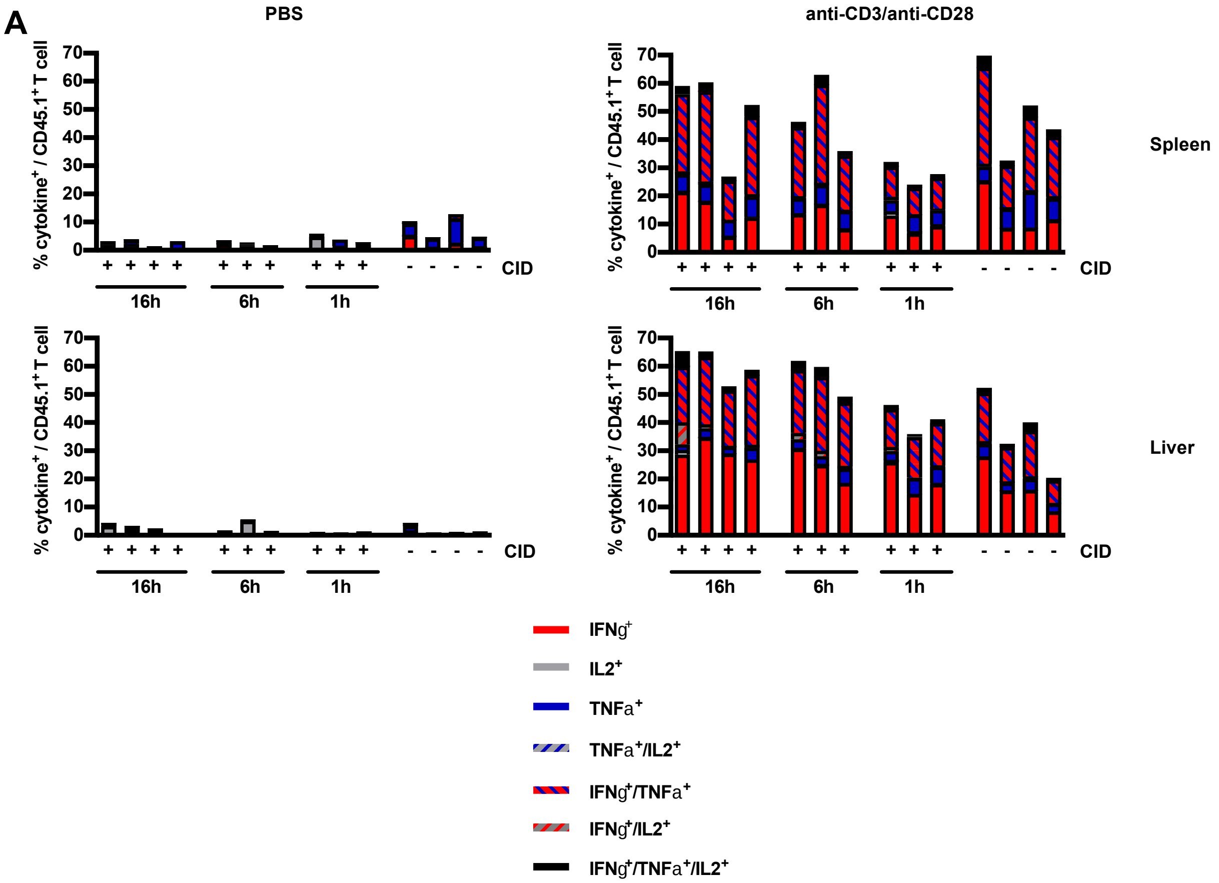

Supplementary Figure 6. Controls of intracellular cytokine production of *in vivo* depletion kinetic. Infection of Rag2$^{-/-}$IL-2Rgc$^{-/-}$ mice with HBV was achieved using an adeno-associated vector. After establishment of a stable infection around 5 weeks later, 2 x 10^6 iC9$^+$-S-CAR$^+$ T cells (n = 14) were administered i.p. per mouse. CID was administered between day 6 and 7 and mice were sacrificed on the same day but at different timepoints after CID administration (1h (n = 3), 6h (n = 3) and 16h (n = 4). 4 additional mice received no CID. **(A)** Ex-vivo functionality of transferred CD45.1$^+$ T cells in the spleen and the liver (B day 7, C day 14) was determined via measurement of intracellular cytokine expression (TNF-α, IFN-γ and IL-2) after overnight-culture on plate-bound HBsAg (Figure 6E,F) for specific restimulation, on plate-bound anti-CD3/anti-CD28 antibodies (right side) for unspecific restimulation and on PBS (left side) as a negative control. (A) Each column represents an individual animal.
